# Targeting cellular senescence in kidney diseases and aging: A focus on mesenchymal stem cells and their paracrine factors

**DOI:** 10.1186/s12964-024-01968-1

**Published:** 2024-12-18

**Authors:** Seyyedeh Mina Hejazian, Seyyed Sina Hejazian, Seyyedeh Mina Mostafavi, Seyed Mahdi Hosseiniyan, Soheila Montazersaheb, Mohammadreza Ardalan, Sepideh Zununi Vahed, Abolfazl Barzegari

**Affiliations:** 1 Kidney Research Center, Tabriz University of Medical Sciences, Tabriz, Iran; 2 Neurosciences Research Center, Tabriz University of Medical Sciences, Tabriz, Iran; 3 Ayatollah Taleghani Hospital, Research Development Unit, Shahid Beheshti University of Medical Sciences, Tehran, Iran; 4 Molecular Medicine Research Center, Tabriz University of Medical Sciences, Tabriz, Iran; 5 Department of Medical Biotechnology, Faculty of Advanced Medical Sciences, Tabriz University of Medical Sciences, Tabriz, Iran

**Keywords:** Cellular senescence, Kidney aging, Chronic kidney disease, Acute kidney injury, Mesenchymal stem cells, Secretome of MSCs

## Abstract

Cellular senescence is a phenomenon distinguished by the halting of cellular division, typically triggered by DNA injury or numerous stress-inducing factors. Cellular senescence is implicated in various pathological and physiological processes and is a hallmark of aging. The presence of accumulated senescent cells, whether transiently (acute senescence) or persistently (chronic senescence) plays a dual role in various conditions such as natural kidney aging and different kidney disorders. Elevations in senescent cells and senescence-associated secretory phenotype (SASP) levels correlate with decreased kidney function, kidney ailments, and age-related conditions. Strategies involving senotherapeutic agents like senolytics, senomorphics, and senoinflammation have been devised to specifically target senescent cells. Mesenchymal stem cells (MSCs) and their secreted factors may also offer alternative approaches for anti-senescence interventions. The MSC-derived secretome compromises significant therapeutic benefits in kidney diseases by facilitating tissue repair via anti-inflammatory, anti-fibrosis, anti-apoptotic, and pro-angiogenesis effects, thereby improving kidney function and mitigating disease progression. Moreover, by promoting the clearance of senescent cells or modulating their secretory profiles, MSCs could potentially reverse some age-related declines in kidney function.

This review article intends to shed light on the present discoveries concerning the role of cellular senescence in kidney aging and diseases. Furthermore, it outlines the role of senotherapeutics utilized to alleviate kidney damage and aging. It also highlights the possible impact of MSCs secretome on mitigating kidney injury and prolonging lifespan across various models of kidney diseases as a novel senotherapy.

## Background

As life expectancy rises and the population ages, there is a concurrent increase in the incidence of chronic diseases [1]. The aging process progressively impacts all body organs, with the kidney being particularly susceptible to these effects [2, 3]. Structural transformations and a gradual deterioration in kidney function are among the biological consequences of kidney aging [4]. A hallmark of aging is cellular senescence, recognized as a major contributor to the processes of aging and age-associated pathologies. The load of these cells increases with age and stress within the kidney.

Cellular senescence is defined as a state distinguished by a modified transcriptome, the secretion of pro-fibrotic and pro-inflammatory factors, and typically irreversible cessation of growth. Factors such as DNA damage, endoplasmic reticulum stress, oxidative stress, mitochondrial dysfunction, and epigenetic regulation can trigger cellular senescence in the kidney cells. While cellular senescence plays essential roles in tissue regeneration, tumor suppression, and embryonic development, the chronic accumulation of senescent cells due to processes such as aging, cellular stress, and other harmful agents contributes to the pathogenesis of age-related diseases, malignancies, metabolic diseases, and kidney diseases [5, 6].

Cellular senescence serves as a crucial link between chronic kidney disease (CKD), and acute kidney disease (AKI), with shared mechanisms involving inflammation, fibrosis, and aging that contribute to kidney dysfunction. The interplay between these conditions underlines the significance of addressing cellular senescence in therapeutic strategies for delaying and mitigating kidney dysfunction and age-related manifestations.

To date, three key experimental strategies are used to manipulate or remove senescent cells: depleting senescent cells, manipulating triggering pathways of senescence (mainly P16^INK4A^ and P21^CIP1^), and targeting senescent cells and/or their secreted factors pharmacologically [7]. Given the similarities between kidney senescence and kidney diseases in terms of mechanism, etiology, pathological changes, phenotype, and outcome, it is speculated that mesenchymal stem cells (MSCs) and their secreted biofactors that reverse or halt AKI/CKD can also delay/prevent kidney senescence as a new therapy. The regenerative effects of MSCs are facilitated by their differentiation capacity to replace damaged tissues, and the paracrine release of factors, such as chemokines, cytokines, extracellular vesicles (EVs) mainly microvesicles and exosomes, and growth factors, called secretome.

Unlike other reviews that may focus solely on the therapeutic effects of MSCs-derived factors in AKI/CKD or cellular senescence independently, this paper specifically highlights the potential of MSC-derived secretome as a novel therapeutic strategy to counteract the senescence, aging, and diseases of the kidney. This dual focus underscores the relevance of MSCs in both mitigating age-related kidney decline and promoting kidney health. Furthermore, it seeks to connect fundamental research with clinical practice, offering valuable insights for future therapies focused on kidney aging and senescence, thereby filling a significant gap in the existing literature.

### Cellular senescence

Similar to differentiation, apoptosis, and replication, senescence can manifest at any stage of life, indicating irreversible growth arrest and resistance to apoptosis. Senescent cells have a distinctive secretome called the senescence-associated secretory phenotype (SASP) including profibrotic and proinflammatory factors with endocrine, paracrine, and autocrine activities [8]. There are also alterations in the morphology of these cells such as abnormal organelles, granularity in the cytoplasm, and flattened cell bodies.

Cell senescence can occur either as replicative senescence (due to the reduced telomerase activity and loss of telomeres) or stress-induced senescence. Stress-induced senescence can be promoted by DNA damage, oxidative stress, reactive oxygen species (ROS), or inflammation [9, 10]. ROS production results in premature senescence by upregulation of p21 and p16 [11]. Immediately after ischemia/reperfusion (I/R) injury, kidney expression of β-galactosidase, a biomarker of elevated autophagy in senescent cells, is induced by an overexpression of p21 [12]. Moreover, both senescence and overexpressed p21 produce TGF-β that leads to fibrosis [13].

Senescence is accompanied by elevated secretion of EVs [14] and these vesicles perform key roles in senescent cells [15, 16], inducing autocrine and paracrine senescence in their adjacent cells [16]. Malfunctioned exosome secretion leads to DNA damage response (DDR) in all cells [17]. The surrounding environment and adjacent cells are also impacted by the SASP and the presence of cytokines, proteases, growth and hemostatic factors, laminin, fibronectin, and collagens (extracellular matrix proteins) [18] .

During the aging procedure, the immune system also becomes dysfunctional, and consequently senescent cells are not removed in time, inducing chronic inflammation and fibrosis [5]. The performance and outcome of SASP can be different based on the factors that cause cell aging. In the chronically induced aging process, it can activate pathogenic reactions through the accumulation of macrophages and the stimulation of malignant cells in the surrounding environment [19]. Instead, in the transient presence of senescent cells, SASP plays a physiological role and exerts a beneficial effect, limiting fibrosis in response to damage by induction of fibroblast senescence, in successful tissue homeostasis and embryonic organogenesis, healing, or repairing [20]. Thus, senescent cells provide a mechanism that avoids the proper proliferation of injured cells but continues to be metabolically active and remains viable.

Another feature of senescent cells is their ability to resist apoptosis. Various mechanisms facilitate the sustenance of senescent cells (Table 1), including the induction of the unfolded protein response, metabolic reprogramming, and strategies for evading the detection of the immune system (reviewed in Ref [21]).

**Table 1:** Key molecular pathways involved in kidney cell senescence

Mechanisms	Signaling pathways	Description	Ref.
**Telomere damage/shortening**	↑Genomic instability and loss of cellular function	A critical marker associated with cellular aging that can exacerbate harmful effects.	[22]
**Persistent activation of DDR**	ATM, ATR, p53, p2	DNA damage activates ATM and ATR kinases that phosphorylate p53, leading to the induction of p21, an inhibitor of CDKs. This event halts cell cycle progression by inhibiting cyclin-CDK complexes.	[23]
**Cell Cycle Arrest**	↑p53/p21 Pathway	Activation of p53 induces p21 expression, inhibiting CDKs, and causing cell cycle arrest.	[24]
	↑p16^INK4a^/Rb Pathway	p16^INK4a^ inhibits CDK4/6, preventing RB phosphorylation, leading to cell cycle arrest.	
**SASP**	↑NF-κB signaling pathway	Activation of NF-κB promotes the secretion of pro-inflammatory cytokines and growth factors.	[25]
	↑IL-6/IL-8 Pathway	The secretion of pro-inflammatory cytokines and profibrotic factors such as IL-1, IL-6, and TGF-β	
**Mitochondrial dysfunction**	↑ROS production ↓AMPK pathway	Mitochondrial dysfunction increases ROS production, causing oxidative damage. ↑Flatted/enlarged shape mitochondria	[26]
**Reduced autophagy**	↓AMPK	The AMPK pathway is integral to maintaining cellular health and longevity by regulating energy metabolism, autophagy, inflammation responses associated with SASP, and oxidative stress responses.	[27]
	↑mTOR Pathway	mTOR inhibition promotes autophagy, which helps in degrading damaged cellular components.	
**Epigenetic changes**	Histone modification pathways	Changes in histone acetylation/methylation alter chromatin structure and gene expression.	[28]
	DNA methylation pathways	Alterations in DNA methylation contribute to the stable maintenance of senescence.	
**ER Stress**	UPR sensors, ATF4/p16	Accumulation of misfolded proteins in the ER triggers UPR signaling pathways that lead to cell cycle arrest, cellular senescence, and age-related diseases.	[29]
**Dysregulated Sirtuins**	↓Sirtuin 1	Sirtuin 1 inhibits the p53/p21 pathway by deacetylating p53.	[30]
**Apoptosis resistance**	↑BCL-2 family pathways	Upregulation of BCL-2 family proteins prevents apoptosis in senescent cells.	[31]
	↑PI3K/AKT pathway	PI3K/AKT signaling promotes cell survival and resistance to apoptosis.	
**Dysfunctional metabolism**	↑AMP/ATP to ADP/ATP ratio	↑ Glycolysis	[32]
**↓Klotho**	↑Wnt/β-catenin signaling ↑TGF-β pathways to mitigate fibrosis	**↓**Klotho is associated with Ischemic stress, cellular senescence, and fibrosis in renal tissues.	[33, 34]

*AMPK* AMP-activated protein kinase*, CDK *Cyclin dependent kinase*, DDR *DNA damage response*, ER *Endoplasmic reticulum*, IGF-1 *Insulin-like growth factor 1*, *mTOR Mammalian target of rapamycin, *PI3K *Phosphoinositide 3-kinase, *Rb* retinoblastoma protein, *ROS *Reactive oxygen species, *SASP *Senescence-Associated Secretory Phenotype, *TGF-β* Transforming growth factor beta, *UPR* Unfolded protein response

### Kidney cell senescence and aging: molecular, cellular, and physiological changes

Cellular senescence has a reparative role in kidney injury and promots tissue repair and regeneration. These cells can secrete various factors that may enhance local inflammation and recruit immune cells necessary for tissue repair. Specific markers, such as p21 and p16, can indicate a protective response that helps limit damage and promote healing after kidney injury. Conversely, persistent senescence can lead to detrimental effects, particularly when senescent cells accumulate over time. This accumulation can contribute to chronic inflammation, fibrosis, and further decline in renal function [35, 36].

**Fig. 1:**
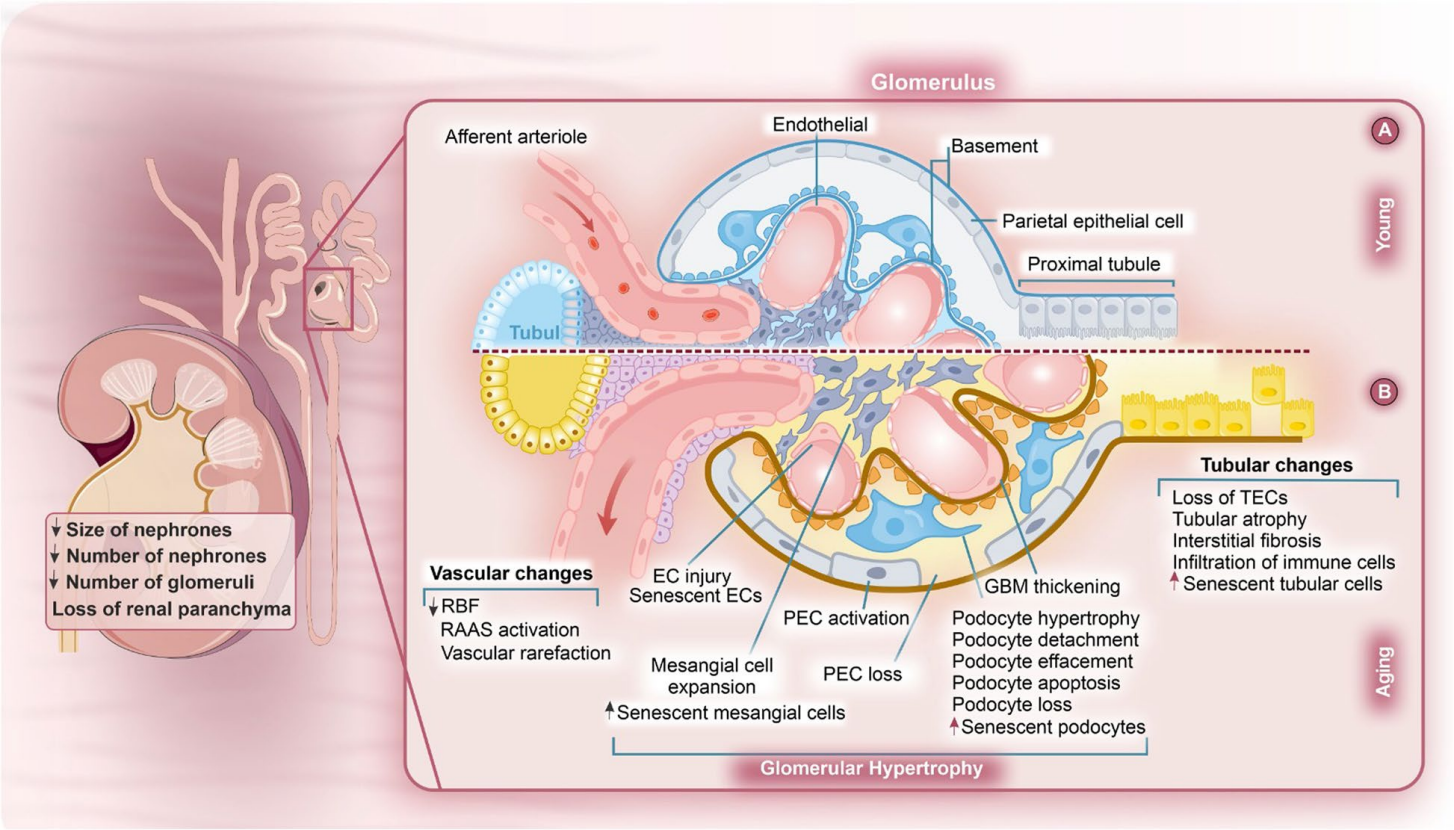
The physiological differences in the aged kidney. The differences between (**A**) younger and (**B**) aged kidney. Renal aging is influenced by various factors including gender, genetic background, race, and pivotal mediators like oxidative and nitrosative stresses, chronic inflammation, RAAS, hormones (sex hormones, Klotho, FGF-23), diminished kidney function and repair capabilities, and underlying cardiovascular conditions. The irreversible and permanent growth arrest of senescent cells, imbalance of proliferation/apoptosis, and reduced repair after organ damage are the central paradigm of kidney aging, decreasing repair after injury, and increasing sensitivity to injury. Vascular changes, glomerular hypertrophy, EC injury, mesangial cell expansion, PEC loss, and tubular changes are presented in the aged kidney. EC, Endothelial cell; FGF-23, Fibroblast growth factor-23; GBM, Glomerular basement membrane; PEC, Parietal Epithelial Cell; RAAS: Renin-angiotensin-aldosterone system; RBF, Renal blood flow; RAAS, Renin-angiotensin-aldosterone system; TEC, Tubular epithelial cells

Various insults trigger the senescence in different kidney cells via distinct pathways. Stress factors, kidney diseases, and physiologically kidney aging can activate cell senescence signaling pathways. Kidney cellular senescence can act as both a cause, through triggering cellular senescence mechanisms, and a consequence of kidney disease, as a result of the progression of AKI and CKD [37]. Cell senescence in kidney cells induces microscopic and macroscopic changes, implying changes in kidney function. The accumulation of senescent cells in the kidneys also leads to structural changes such as glomerulosclerosis and tubular atrophy, common in aging kidneys (Fig. 1). In aged populations, AKI increases the risk of progression to CKD, underscoring a significant association between acute injury and chronic disease via mechanisms that encompass cellular senescence [36]. In the following sections, we will first cast light on the current understanding of the molecular mechanisms involved in kidney cell senescence, and then elucidate the mutual interactions between cellular senescence, kidney diseases, and aging.

### Kidney tubular cell senescence

**Fig. 2:**
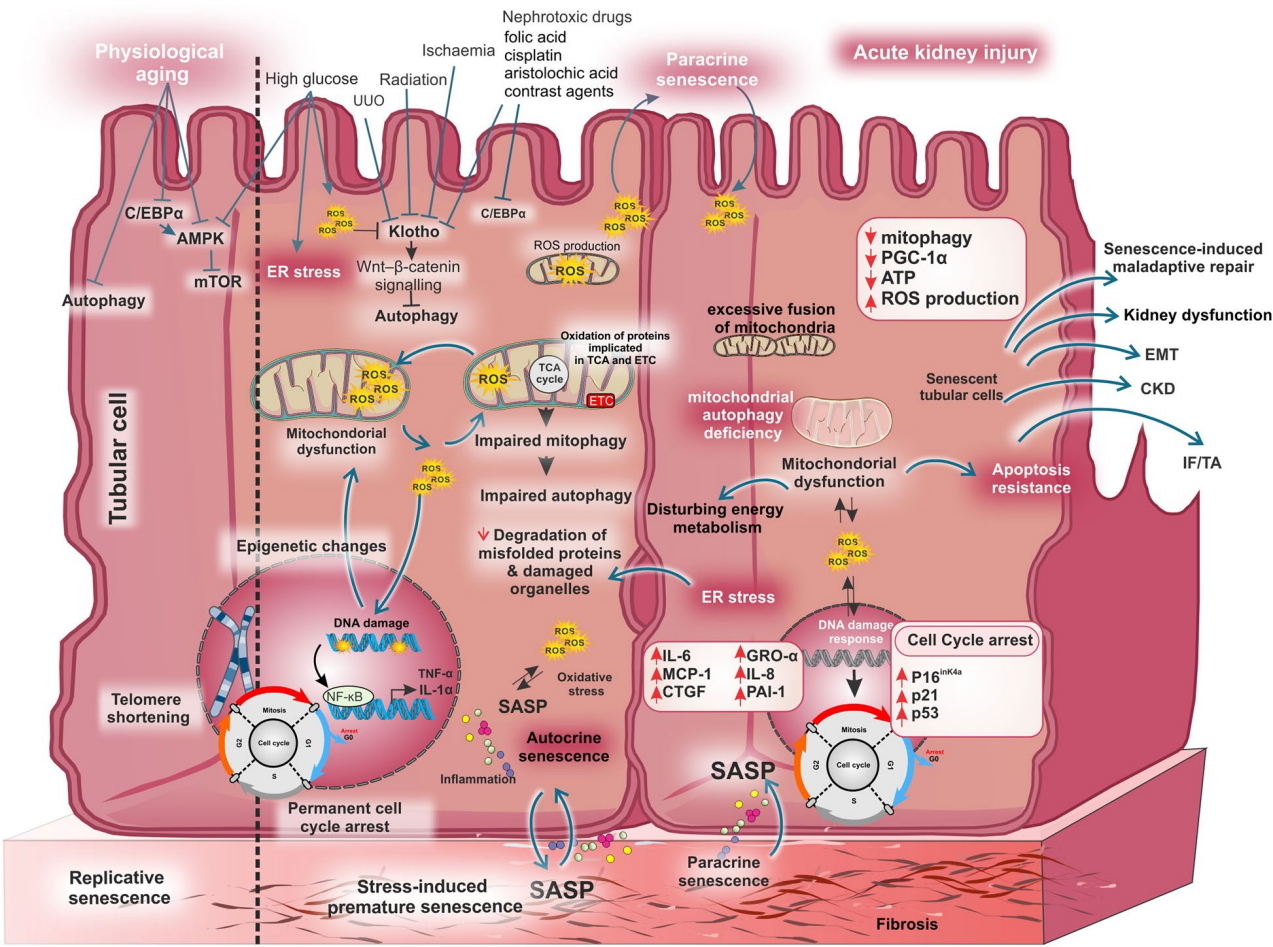
The mechanisms involved in the kidney tubular cell senescence. Replicative (telomere shortening) and stress-induced premature senescence in the kidney tubular cells are shown in detail. Different stimuli (ischemia/reperfusion, high glucose, radiation, UUO, cisplatin, folic acid, aristolochic acid, contrast agents, etc.) induce the TEC senescence through different pathways mainly DNA damage, increased levels of intracellular ROS, epigenetic changes, cell cycle arrest, decreased levels of klotho, mitochondrial dysfunction, impaired autophagy, and ER stress. In terms of dynamics, structure, and function, different alterations can be seen in senescent cells’ mitochondria. Mitochondria are elongated and hyperfused, mitochondrial protein leaks, and the metabolites of the TCA cycle are increased in senescent cells. Senescent cells accumulate dysfunctional mitochondria and conversely mitochondria by producing ROS and pro-inflammatory phenotype result in cellular senescence. Moreover, ATP/ADP and NAD^+^/NADH ratios, and membrane potential are decreased in senescent cells’ mitochondria. Additionally, decreased mitophagy increases dysfunctional mitochondria, producing high ROS and DAMPs. High levels of mitochondrial ROS lead to the oxidation of DNA, lipids, and proteins, causing DNA breaks, mainly at telomere regions. Activation of NF-κB (a major regulator of the SASP) by direct or indirect impact of ROS engages pro-inflammatory pathways, resulting in senescence. AMPK, AMP-activated protein kinase; ATP, Adenosine triphosphate; C/EBPα, CCAAT/enhancer-binding protein alpha; CKD, Chronic kidney disease; CTGF, Connective tissue growth factor; EMT, Epithelial–mesenchymal transition; ER: Endoplasmic reticulum; ETC, Electron transport chain; IF/TA: Interstitial fibrosis/ tubular atrophy; GRO-α, Growth regulated alpha; MCP-1, Monocyte chemoattractant protein-1; mTOR, Mammalian target of rapamycin; NF-κB, Nuclear factor kappa-light-chain-enhancer of activated B cells; PAI-1, Plasminogen activator inhibitor-1; PGC-1α, Peroxisome proliferator–activated receptor gamma coactivator-1 alpha; ROS, Reactive oxygen species; SASP, Senescence-associated secretory phenotype; TCA, Tricarboxylic acid; TNF- α, Tumor necrosis factor; UUO, Unilateral ureteral obstruction

Tubular epithelial cells (TECs) are considered the main location for kidney senescence and aging, exerting a significant impact on the physiological and pathological functions of the kidneys. These cells have abundant mitochondria, indicating their heightened energy and metabolic requirements. Kidney TECs are predominantly located in regions of the kidney with restricted blood flow and oxygen availability, making them intrinsically more susceptible to injury, reviewed comprehensively elsewhere [38]. Different insults such as ischemia, high glucose, radiation, nephrotoxic drugs, and contrast agents result in tubular cell injury and senescence by the disruption of the cytoskeleton, degradation of DNA, and damage in TEC’s membrane, culminating in tubular necrosis, apoptosis, and ferroptosis. Additionally, under AKI, TECs are susceptible to oxidative stress, mitochondrial dysfunction, and disruptions in energy metabolism and autophagy, leading to stress-induced cellular senescence (Fig. 2). Hyperglycemia by triggering endoplasmic reticulum stress through activating ATF4-p16 signaling [29] and sodium-glucose cotransporter 2 (SGLT2) /p21-dependent pathway [39] can trigger tubular senescence. Likewise, SGLT2 is activated by hedgehog interacting protein, promoting TEC senescence in a type 1 diabetes model [40]. TEC senescence can be also induced by the overexpression of Wnt9a [41], activation of the Wnt–β-catenin pathway [42], and inhibition of AMPK–mTOR signaling [43]. Moreover, TEC senescence is regulated by Myd88 through Toll-like receptor signaling [6]. The senescence of TECs correlates with a decline in the rate of renal recovery after kidney injury, maladjusted repair, kidney dysfunction, and development of CKD, and is accompanied by renal fibrosis progression [44].

### Glomerular podocyte senescence

Podocytes are terminally differentiated kidney cells that are crucial in maintaining the proper functioning of the filtration barrier within the glomerulus. Podocyte senescence is a process implicated in kidney injury and aging. According to some research, podocyte damage induces senescence, thereby hastening glomerular aging in young mice and cultured cells [45]. Floge et al. initially identified the pivotal role of podocytes in age-related glomerulosclerosis [46]. Research on human beings has further emphasized the significance of podocytes as a crucial cell type impacted by aging since advanced age presents itself by podocyte depletion in people lacking evidence of renal pathology [47].

**Fig. 3:**
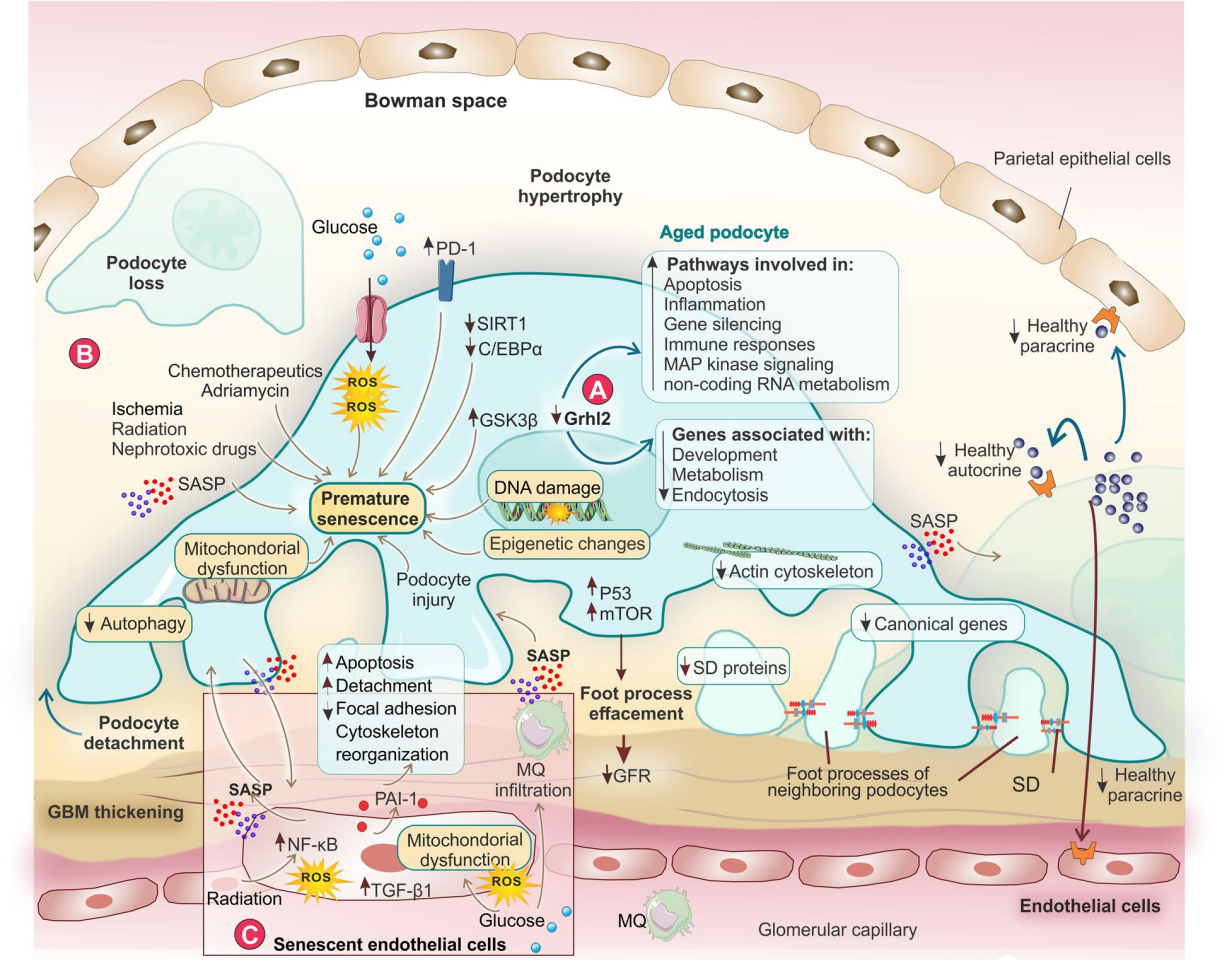
The mechanisms involved in podocyte and endothelial cell senescence. **A** podocyte-specific factors (transcription factors like Grhl2) lead to aged podocyte. **B** Environmental, systemic, and common aging-related factors cause premature podocyte senescence via different mechanisms. **C** Different factors such as CKD, radiation, and high level of glucose play a critical role in the development of senescent phenotype in endothelial cells. PAI-1 as a mediator in the communication between endothelial and podocyte cells has a role in the formation of glomerular lesions in the aging process in both murine and human subjects. Within this framework, the initiation of a senescence regimen in endothelial cells proves to be indispensable. Senescent glomerular endothelial cells by expression of PAI-1 drive podocyte damage by promoting the reorganization of the F-actin cytoskeleton, decreasing the number of focal adhesions, and stimulating podocyte apoptosis and detachment. C/EBPα, CCAAT/enhancer-binding protein alpha; GBM, Glomerular basement membrane; GFR, Glomerular filtration rate; GSK3B, Glycogen Synthase Kinase 3 Beta; MQ, Macrophage; mTOR, Mammalian target of rapamycin; NF-κB, Nuclear factor kappa-light-chain-enhancer of activated B cells; PAI-1, Plasminogen activator inhibitor-1; PD-1, Programmed cell death protein 1; ROS, Reactive oxygen species; SASP, Senescence-associated secretory phenotype; SD, Slit diaphragm; SIRT1, Sirtuin-1; TGF-β, Transforming growth factor beta Grhl2; Grainyhead-like2

A combination of environmental, systemic, common aging-related factors [hypertension, diabetes, and obesity], and podocyte-specific factors [alterations in definite transcription factors such as Grhl2 (Grainyhead-like2)] contribute to podocyte senescence (Fig. 3A-B). Senescence signaling pathways in podocytes involve key mediators like p16^INK4A^, p21, and p53, influencing cellular senescence, degeneration, and the expression of fibrogenic factors [48, 49]. Besides cellular senescence, the phenotype of aging podocyte is distinguished by alterations in ultrastructure and functionality, oxidative, cellular, and endoplasmic reticulum stress, and hypertrophy, alongside diminished autophagy, and the heightened manifestation of genes associated with aging.

The downregulation of sirtuin 1 and C/EBPα has been identified in the aged podocytes. Sirtuin1 is a gene associated with longevity that serves to protect podocytes from inflammation and oxidative stress and controls the function of numerous transcription factors. C/EBPα is a transcription factor that manages autoimmunity, inflammation, and energy metabolism in podocytes. C/EBPα deficiency in podocytes has been shown to aggravate podocyte senescence and kidney damage through the AMPK/mTOR pathway (mechanisms associated with metabolism) in aging mice. On the other hand, the upregulation of podocyte-specific programmed cell death 1 (PD-1) and glycogen synthase kinase 3β (GSK3β) is connected with the senescence of podocytes, giving rise to a decline in the function and histology observed with age. The upregulation of PD-1 in aged podocytes evades detection by immune cells, affecting surrounding cells through the SASP [50]. GSK3β and PD-1 might regulate senescent podocytes via a comparable pathway since GSK3β is a pivotal upstream kinase, regulating PD-1 expression [51]. Beyond aging, ischemia, radiation, nephrotoxic drugs, and unilateral ureteral obstruction (UUO) can induce podocyte senescence through the inhibition of C/EBPα, reduction in AMPK-mTOR signaling, and activation of Wnt-β-catenin signaling, which inhibits autophagy (Fig. 3A, B). In the aging kidney, podocyte senescence has been connected with the induction of glomerulosclerosis [52].

### Glomerular endothelial cell senescence

The notion of vascular senescence and early vascular aging stands as a crucial risk factor for the premature emergence of cardiovascular complications. The senescence of glomerular endothelial cells plays a critical role in age-related kidney disease, particularly in the development of glomerulosclerosis through mechanisms involving plasminogen activator inhibitor-1 (PAI-1) [53]. TGF-β1 can also induce nuclear translocation of p16, contributing to endothelial cellular senescence in the glomeruli [54]. As research indicates, endothelial cells and macrophages are prominent sources of SASP components in hyperglycemic renal tissue [55]. High glucose levels can cause macrophage infiltration, the activation of NOX1-PKC signaling, and mitochondrial dysfunction, culminating in increased ROS levels and senescence of endothelial cells. Radiation triggers the onset of cellular senescence in glomerular endothelial cells by stimulating the NF-κB signaling [56], accumulation of M1 macrophage, and increasing ROS and p38 MAPK signaling [57]. Angiopoietin-1 is reported to prevent H_2_O_2_-induced glomerular endothelial cells by the ERK1/2 pathway [58]. Aged podocytes may stimulate alterations in the endothelial cells and vice versa. Senescent glomerular endothelial cells drive podocyte damage by promoting the F-actin cytoskeleton reorganization by expression of PAI-1, reducing the number of focal adhesions, and stimulating podocyte apoptosis and detachment [53], (Fig. 3C).

Hypertrophy of the intima and media, sclerosis of the vascular wall, and the formation of atheromatous plaques are structural changes in the renal vessels due to aging, similar to the changes observed in vessels in other organs of the body. Increased tortuosity and loss of glomerular integrity, creation of direct shunt between afferent and efferent vessels, hypertrophy of the vascular wall, and reduction of the lumen diameter of afferent arteries are changes that occur mainly in the aging processes [59].

### Senescence in other kidney cells

Kidney mesangial cells exert a central role in preserving the function and structure of the glomerulus. High levels of glucose induce mesangial cell senescence via AGE-STAT5 signaling, inhibiting autophagy, and the accumulation of injured mitochondria and ROS. Senescence of kidney scattered tubular-like cells is induced by ischemic renovascular that weakens their reparative capacity [60]. In a study on renal transplant biopsies, all cases had a positive P16^INK4A^ nucleus staining in their distal tubules and collecting ducts, accompanied by vascular smooth muscle cells (VSMCs), parietal epithelium of glomeruli, podocytes, and interstitial cells positive staining in some cases [61].

### Mutual interaction between kidney cell senescence and AKI

Life-threatening AKI is mainly triggered by surgeries, ischemia, and nephrotoxic stimuli [34, 62]. During the occurrence of AKI, the kidney is subjected to various forms of stress and challenges [38, 63], which can readily induce cellular senescence. There is a mounting body of evidence indicating a close association between cellular senescence and the underlying pathophysiology of AKI, as they both exhibit a cell cycle arrest at the G2/M phase, heightened oxidative stress, an increase in cyclin-dependent kinase inhibitors, telomere shortening, and an exacerbated in the fibrotic processes. The reduction of klotho expression in the context of AKI might also facilitate the onset of senescence and hinder the recovery mechanisms following an episode of AKI [64]. Furthermore, the activation of Notch signaling has been observed in I/R-induced AKI models, subsequently triggering the activation of p21 and p16^INK4a^ and intensifying interstitial fibrosis development [65]. On the other hand, emerging evidence indicates that senescent TECs contribute to the development of AKI and the transition from AKI to CKD [66, 67].

In cases of mild and moderate AKI, approximately 70% of quiescent tubular cells can re-enter the cell cycle from a dormant state within the first 24 h, engaging in regeneration and proliferation, thereby facilitating the recovery of kidney function and structure [68]. However, during maladaptive repair after severe AKI, TECs can undertake a senescence-like phenotype due to telomere shortening, elevated levels of cyclin kinase inhibitors (mainly p21), and downregulated in Klotho expression. In a paracrine manner, senescent TECs give rise to senescence in surrounding cells [69]. The profibrotic and proinflammatory mediators of SASP stimulate the infiltration of immune cells and tubular cell damage generating in persistent tubulointerstitial inflammation, the proliferation of fibroblasts, and extreme extracellular matrix deposition augmenting kidney damage and the progression to CKD. Senescent immune cells, such as CD14^+^ CD16^+^ monocytes and CD28^−^ T cells also exacerbate kidney ROS production and chronic inflammation, promoting CKD progression [44].

The selective removal of senescent cells significantly alleviates physical dysfunction and a healthy lifespan in mice. In a contrast-induced AKI model, paricalcitol pre-treatment could reduce tissue damage and cellular senescence [66]. Likewise, Lipoxin A4 by blocking crosstalk between premature senescence and inflammation could restore kidney function in septic-induced AKI mice [67]. These data support that cellular senescence can be a new target for AKI management and restoring its balance could have prospective benefits.

### Mutual interaction between kidney cell senescence and CKD

CKD serves as a clinical model of premature and enhanced kidney aging. Individuals with CKD experience a substantially hastened aging process described by cardiovascular diseases, osteoporosis, muscle wasting, persistent uremic inflammation, and frailty, even before reaching end-stage renal failure. All of these complications share similar pathological features associated with senescent cells. However, due to the complex correlation between CKD and senescence, it is challenging to judge if the senescent kidney cells are a consequence or a trigger of CKD.

Evidence indicates that kidney senescence participates in the progression and pathogenesis of CKD. For example, in kidney allografts undergoing the transition from AKI to CKD, the level of p21 is elevated [70]. Moreover, in an elderly I/R injury mouse model of CKD, microvascular rarefaction and inflammation are more significant compared to young controls [71]. Increased levels of senescence markers and senescent cells have been documented in the I/R injury and UUO models, as well as in kidney biopsy from patients with CKD [72]. In these studies, senescent cells were predominantly identified as tubular epithelial cells [42], implying that kidney diseases progress in the context of senescence within these specific cells.

The CKD-associated activation of anti-aging (e.g. reduced expression of klotho, loss of autophagy) and aging-promoting (e.g. inflammation, oxidative stress, uremic toxins, overactivation of the RAS, hyperphosphatemia) factors make important contributions to an elevated cellular senescence in CKD. The SASP in senescent cells shares similarities with the CKD-associated secretory phenotype [35]. Both involve the secretion of profibrotic and pro-inflammatory factors that can lead to further tissue damage and dysfunction. For instance, in a renal fibrosis model following I/R injury, higher levels of TNF-α and MCP-1 were reported [71]. Moreover, elevated levels of MCP-1, epidermal growth factor, IL-1α, IL-6, and VEGF are seen in the blood samples of patients with early CKD [73]. Additionally, urine studies of CKD patients were in favor of elevated TGF-β1, MCP-1, and IL-8 [74]. Cellular senescence in aged kidneys with CKD shows reduced function and elevated vulnerability to AKI [71, 75–78].

### The role of senescence in glomerular diseases

Senescence of TECs is also involved in glomerular diseases including diabetic nephropathy, IgA nephropathy (IgAN), UUO, focal segmental glomerulosclerosis [79], and lupus nephritis (LN) [80]. Moreover, studying tubular TECs of healthy living kidney donors and patients with glomerular disease using P16^INK4A^ staining revealed that senescence was more present in cases compared to controls (80% vs. 21%) [80]. Furthermore, kidneys with IgAN are associated with elevated P21^CIP1^ and P16^INK4A^ protein expression in tubular cells [72]. Studies showed that telomere shortening and aging biomarkers especially accompanying the progression of IgAN were not significant in other glomerular diseases [81]. Renal TECs in patients with IgAN displayed features of augmented senescence similar to mechanisms accompanying normal aging [72]. Cellular senescence has a key role in LN pathogenesis by accumulating p16^INK4a^-positive cells [82]. There is a clear association between the accumulation of this type of cells in the biopsy of all patients with lupus and the severity of kidney involvement [83].

Senescent podocyte, endothelial, and mesangial cells may contribute to DN. Recent studies have shown that proximal TECs are the main target for diminished glucose-induced metabolic disarrays in DN. High glucose levels by increasing miR‐378i expression, an impending biomarker of renal impairment, can prompt the senescence of TECs. In the tubular, meningeal, and podocyte cells of patients with type 2 diabetes, the level of p16 expression and senescence-associated beta-galactosidase (SA‑β‑gal) activity is increased, establishing a straight connection between hyperglycemia and the initiation of senescence [84].

### The role of senescence in metabolic-associated kidney diseases

The kidney has various metabolic roles, hence, metabolic syndrome has important effects on the kidney by aggravating kidney damage [85]. It has been shown that the intensity of allograft, diabetes, and IgAN is correlated with the severity of the senescence [72, 86, 87], which is seen in all of these diseases [72, 88]. However, diabetes is the most known condition in terms of cellular senescence in the renal system. Furthermore, the degree of senescence before kidney transplantation is capable of predicting the patients’ outcome regarding graft success [89], indicating that targeting senescent cells may be an efficient therapeutic strategy in renal diseases, and various studies have reported improved renal function and attenuated kidney fibrosis following decreased senescent cells. For instance, inhibition of p53 based on small interfering RNAs is related to reducing cellular senescence and renal fibrosis in rats [90]. This is believed to be partially in association with the function of cell cycle-arrested tubules (at G2/M) that have a part in the production of increased amounts of renal fibrosis-related [91] SASP components such as TGF-β and CCN2 [92, 93]. Renal exposure to hyperglycemic conditions in patients with DM2 drastically enhances the burden of cellular senescence, which is particularly seen among TECs and podocytes [87]. In one study, seven days of exposure to streptozotocin-induced hyperglycemia in mice caused an increase in the renal burden of cellular senescence through SASP [55]. In another study, an increase in aggregation of senescent cells in hyperglycemic rats took only 10 days, which was mostly seen in cortical cells [94]. Surprisingly, the decreased burden of cellular senescence in an obesity-induced metabolic dysfunction model improved glucose homeostasis and insulin sensitivity that was accompanied by declined microalbuminuria and enhanced podocyte function [95].

### Senescence and kidney fibrosis

Senescent epithelial cells initiate maladaptive repair following aging and injury, contributing to the development of kidney fibrosis [44]. In addition, the extent of renal fibrosis in mice is directly related to the level of senescence markers [71]. Although the exact underlying mechanism is not clear, multiple studies have supported the concept of senescence’s role in renal fibrosis progression through transgenic or pharmacological elimination of senescent cells in animal models [96–98]. The investigation of post-injury renal tissue has revealed senescence-mediated progressive fibrosis, decreased vascular density, and organ malfunction through SASP activation. Moreover, the elevated levels of senescent cells were related to poorer renal function and outcome [99]. This is in line with the previously found evidence that indicates prolonged exposure to SASP (such as stemness induction) impairs tissue regeneration [100].

The role of senescence in kidney fibrosis has been approved by a decline in fibrosis in mice lacking p16 expression after I/R injury [41, 101]. Although most of these studies are animal studies, some studies have indicated the role of senescence in human renal fibrosis. Multiple reports have shown that the burden of senescent cells predicts the transplant renal function and is positively associated with the extent of tubular atrophy and fibrosis [89, 102–104].

### Senescence countering strategies

Given the scenario of kidney cell senescence in kidney aging and diseases, the main question is how to avoid the senescence process and whether AKI and CKD can be repaired. Targeting anti-aging signaling pathways by interventional strategies can be clarified for reversing and/or avoiding cellular senescence over time. These interventions could be alterations in lifestyle [105], modulating SASP, probiotics, antioxidants, inhibitors of NF-κB and mTOR, and activators of Nrf2, sirtuins, and AMPK, as well as senolytic drugs. Maique indicated that klotho inhibits aging induced by high phosphate in the proximal tubular epithelial cells [106]. In another study, klotho could also inhibit mitochondrial cellular aging, DNA injury, and oxidative stress in a mouse with immune complex-mediated glomerulonephritis and thereby increase animal survival, maintain kidney function, improving tubulointerstitial and glomerular injury [107]. Therefore, it is suggested that klotho supplementation may serve as a proper therapeutic approach for the management of CKD and other age-related diseases [108].

**Fig. 4:**
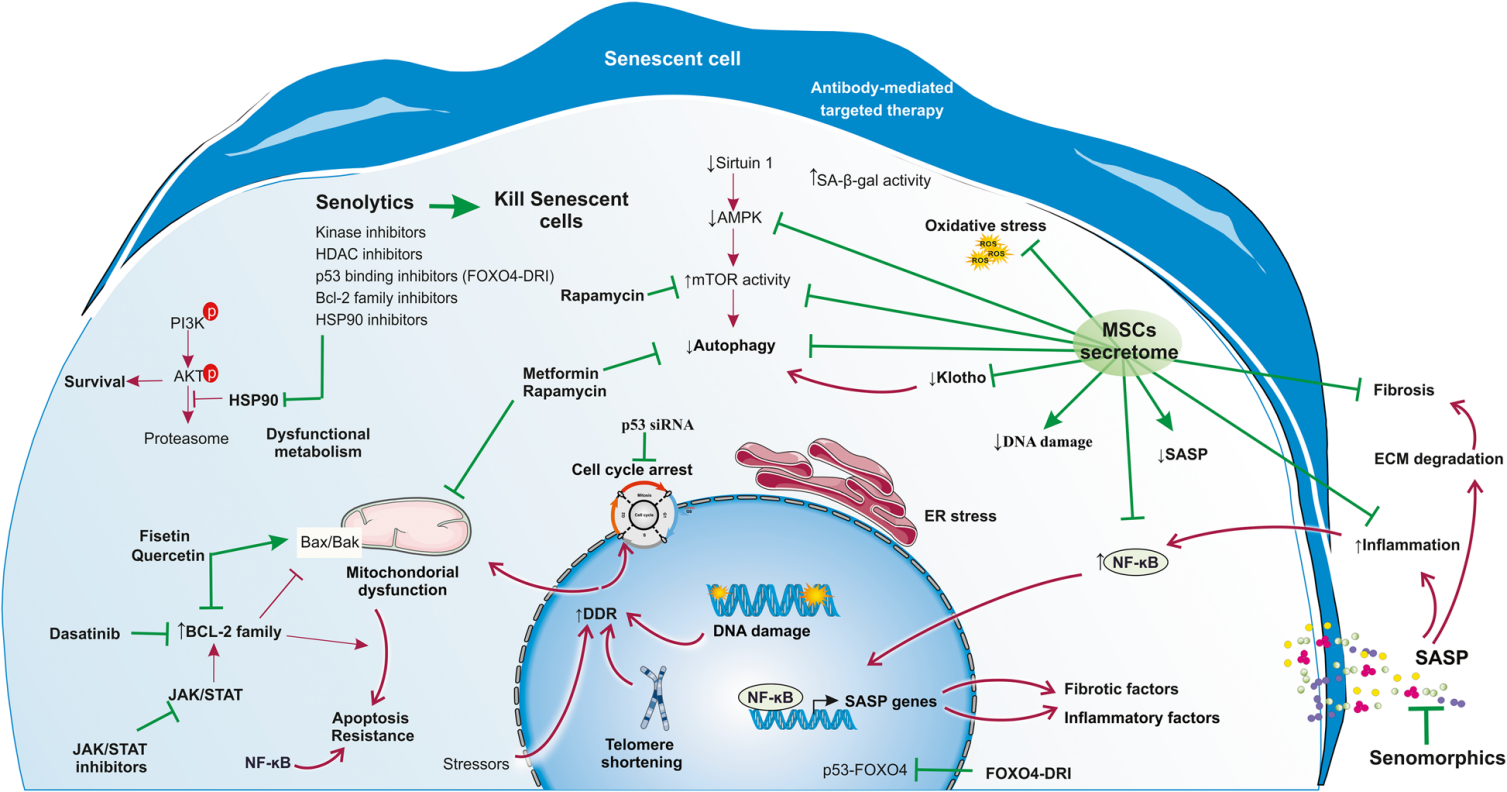
Potential senolytic and senophorphic agents can target different signaling pathways involved in cellular senescence. **A** Senolytic agents are selective killers of senescent cells. They are classified into kinase inhibitors, histone deacetylase (HDAC) inhibitors, heat shock protein 90 (HSP90) inhibitors, p53 binding inhibitors, Bcl-2 family inhibitors, and UBX0101. Senomorphics are blockers of SASP. **B** MSCs and their secreted factors (called secretome) can be considered senotherapeutic biofactors since they can target the main signaling pathways involved in cellular senescence. See the main text for more details. AMPK, AMP-activated protein kinase; DDR, DNA damage response; ECM, Extracellular matrix; ER, Endoplasmic reticulum; FOXO4-DRI, Fork head box O transcription factor 4-D-Retro-Inverso; HSP90, Heat shock protein 90; JAK, Janus kinase; MSCs, mesenchymal stem cells; NF-κB, Nuclear factor kappa-light-chain-enhancer of activated B cells; PI3K, Phosphoinositide 3-kinase; ROS, Reactive oxygen species; SA-β-gal, Senescence-associated beta-galactosidase; SASP, senescence-associated secretory phenotype

Senotherapeutic agents are useful for attenuating aging and age-related disease footprint. These agents are categorized as senolytics (selective killers of senescent cells), senomorphics (blockers of SASP), and senoinflammation (immunity-mediated removers of senescent cells, also called inflammaging) [109]. MSCs and their paracrine factors can be other sources of anti-senescence interventions that modify the characteristics of senescent cells or delete them (Fig. 4).

### Senolytics

The first senolytic agents including Quercetin and Dasatinib were introduced by Zhu et al. in 2015. Quercetin, a plant flavonoid effective, was used against senescent human bone marrow-derived MSCs (BM-MSCs) and endothelial cells of mice, both of which led to a decrease in senescent cell burden [110]. It was shown that Dasatinib and Quercetin treatment could inhibit several age-associated mice disorders including CKD [111] and it has been applied in a human clinical trial with unpublished results (NCT02848131) [7, 112]. Since then, seven types of senolytic agents have been introduced so far, consisting of kinase inhibitors, histone deacetylase (HDAC) inhibitors, heat shock protein 90 (HSP90) inhibitors, p53 binding inhibitors, Bcl-2 family inhibitors, and UBX0101 [113]. In addition, it was reported that oral apigenin could diminish increased expression levels of SASP and IκBζ in old rat kidneys [114].

Senostatics can prevent elements of the senescent phenotype without removing the cells. Drugs familiar to nephrologists including rapamycin (sirolimus) and metformin by improving mitochondrial function and activating autophagy can extend lifespan [115]. Metformin also decreased aging in the TECs [116].

Natural compounds.

Quercetin, fisetin, piperlongumine (PL), and natural phytochemicals present senolytic potentials in in vitro studies. In senesced human WI-38 fibroblasts, PL selectively induces apoptosis through ionizing radiation, H-Ras activation, or replicative exhaustion [117]. Fisetin preferentially removes irradiation-induced senescent HUVECs. However, no effect on IMR90 or pre-adipocytes has been established [118].

### UBX0101

UBX0101 is a small molecule with senolytic potential that inhibits the interaction between MDM2 and p53 [119]. Intra-articular injection of this agent has been associated with decreased accumulated senescent cells in the synovium and articular cartilage of aged mice induced by apoptosis, leading to attenuated osteoarthritis (OA) following trauma [119]. UBX0101 is also capable of improving the cartilage-rejuvenation capacity of chondrocytes within human OA tissue [119]. The first senolytic agent, UBX0101, has entered a phase-1 clinical trial (https://clinicaltrials.gov/ct2/show/NCT03513016).

### P53 binding inhibitor

Forkhead box protein O4 (FOXO4) is a key protein with a pivotal role in keeping senescent cells alive. Bioinformatic studies of RNA sequences of senescent cells induced by I/R suggested that FOXO4-DRI (fork head box O transcription factor 4-D-retro-inverso) peptide is a novel agent with senolytic potential that blocks the interplay between p53 and FOXO4 in senescent IMR90 cells and HUVECs leading to apoptosis in these cells [97]. FOXO4-DRI is also effective in fitness, hair density, and kidney function improvement in aged mice [97].

Although, there is a wide variety of target proteins for senolytic agents for instance p53, Bcl-xL, Bcl-2, HSP90, and a tyrosine kinase, the discovery of novel targets for these agents is of great importance. Moreover, the cell-specific activity of senolytics suggests that cellular senescence is differentially regulated within different types of cells making it difficult to develop a single effective agent for all types of cells [109].

### Senomorphics

Senomorphics are diverse agents with regulatory features in senescent cells with indirect induction of apoptosis. Treatment of senescent cells with senomorphics modifies the phenotype conversion of these cells to young normal cells through SASP, senescence-related signal pathways, and interference with senoinflammation. Among well-known senomorphic compounds, telomerase activators [120], caloric restriction diets [121] and mimetics [122], proteasome activators [123], autophagy activators [124], antioxidants [125], mTOR inhibitors [126], sirtuin activators [127], and anti-inflammatory agents targeting inflammaging /senoinflammation [128] can be mentioned.

ssKU-60,019 induces the practical recovery of the autophagy/lysosome axis, accompanied by metabolic replanning and restoration of mitochondrial function [129]. This agent is an inhibitor of Ataxia-telangiectasia mutated (ATM) kinase activated through DNA double-stranded breaks and is involved in senescent cell regulation [130].

JH4 is another small molecule with senomorphic features that interferes with the junction between progerin and lamin [131]. Progerin is a shortened lamin protein related to the Hutchitson-Gilford progeria syndrome (HGPS) which accumulates by cumulative shortening of telomere throughout fibroblast cells senescence [132]. JH4 could alleviate the deformation of the nucleus and cellular senescence, and return aging biomarkers like growth arrest SA-β-Gal activity, and HGPS [131]. The administration of JH4 significantly reduced multiple age-related disorders [131]. Plant-derived natural compounds, consisting of quercetagetin 3,4′-dimethyl ether [133], (−)-loliolide [134], quercetin-3-O-β-D-glucuronide [135], and juglanin [136] are recognized as agents with senomorphic potential that decline the level of p53 and SAβG in senescent HUVECs and HDFs.

mTOR activity significantly increases in normal senescent kidneys. Although activated mTOR increases p21 expression levels followed by SASP release and cell cycle arrest in senescent cells, rapamycin could block the proinflammatory phenotype as a senomorphic agent [137].

### Senoinflammatory mediators

Senoinflammation (also called inflammaging) indicates the long-lasting, sterile, low-grade, and unresolved inflammatory situation accompanied by aging [138, 139]. NF-κB present in an active form in the elderly, is a principal transcription protein involved in senoinflammation and its activity is connected to common aging regulators, including DNA damage, mTOR, SIRT, FOXO, and IGF-1 [140]. As a result, the prevention of NF-κB has been proposed as a probable target of senomorphics. A peptide with IKK-inhibiting features, the NF-κB-activating kinase, decreased cellular senescence [141]. Furthermore, SASP has also a prominent part in the senoinflammation of senescent cells. Thus, targeting SASP in these cells could be a feasible target of senomorphics. The Janus kinase (JAK)/STAT pathway can modulate SASP and drugs with inhibitory effects on JAK alleviate senoinflammation in senescent cells, mitigate age-related organ malfunction, and improve physical activity in elder mice [142].

Not only are tissue senescent cells capable of contributing to both innate and acquired immunity, but also immune responses clear senescent cells form tissues, maintaining tissue homeostasis mostly via the DNA damage feedback [143]. However, inadequate removal of senescent cells as a consequence of senescent immunosurveillence results in facilitated aggregation of senescent cells in aged tissues and ARDs [143]. Dipeptidyl peptidase 4 (DPP4) is an enhanced glycoprotein in the plasma membrane of senescent fibroblasts, resulting in antibody and NK cell-mediated removal of DPP4-positive senescent cells [144]. CD4^+^ T cells, macrophages, and NK cells also play a vital role in the elimination of senescent cells, malignancy development prevention [144], and embryonic development [145].

Antibody-mediated targeted therapy of senescent cells is an alternative immunotherapeutic strategy to fight senescence. Since CD9 is increased in the plasma membrane of senescent cells [146], CD9 antibody-conjugated nanoparticles are developed to selectively deliver drugs to senescent cells and suppress their phenotype in HDFs [147, 148]. Despite the discovery of new senescent cell-specific cell surface proteins with senescent cells-targeting potential, the essence of new proteins’ discovery is inevitable, whose identification would help improve the development of new strategies for immunity-mediated elimination of senescent cells.

### Targeting kidney cells

Senolytic agents carried by nanomedicines (e.g. conjugates and liposomes) have great potential for targeting and treatment of aged kidney cells especially proximal tubular cells [149]. Some studies reported that megalin and folate receptor 1α could be beneficial for targeting the proximal tubular cells [150, 151]. Small interfering RNA (siRNA) might also be another therapeutic agent against aged kidneys. Molitoris showed that the intravenous injection of p53 siRNA decreases cellular p53 and apoptosis in ischemic- and cisplatin-induced AKI models [90]. Other kidney cells (i.e. podocytes, mesangial [152, 153], and endothelial cells) have also the potential to be targeted via other surface receptors (using liposomes or nanoparticles) [154–156].

### Mesenchymal stem cells and their secreted factors

MSCs are multipotent cells with proliferative, anti-inflammatory, anti-oxidative, antimicrobial, antifibrotic, antitumor, and proangiogenic effects, contributing to tissue homeostasis and regeneration. Only recently has the clinical importance of MSCs therapy for aging commenced [157]. Preclinical and clinical studies have revealed the beneficial effects of MSC therapy in different kidney diseases [158–160]. Due to the engraftment of MSCs, recent evidence suggests that major trophic effects of MSCs are attributed to their paracrine biofactors [161]. Owing to the cell-free sources, MSC-derived products have numerous advantages over MSC therapy including low immunogenicity, no risk of tumor formation, and easy transfer into recipient cells.

MSC-derived EV therapy can improve renal outcomes in multiple animal models of AKI and CKD (Reviewed in Ref [162, 163]). The renoprotective, regenerative, anti-apoptotic, antifibrotic, anti-inflammatory, mitochondrial hemostasis, and immunomodulatory effects of MSC-EVs have been reported in these studies [161] that are mediated by a variety of mechanisms [158]. Moreover, MSC-EVs can inhibit cell apoptosis, stimulate tubular epithelial cell proliferation, and recover kidneys in plenty of AKI and CKD models, indicating their regenerative effects [158–160, 164]. Furthermore, MSC-derived factors can modulate the senescent phenotype and its associated secretory profile by targeting cellular senescence in kidney cells [165]. In the following sections, we highlight the role of MSCs and their products on senescent and aging kidneys during AKI and CKD.

### Targeting cellular senescence by MSCs and their derivatives during AKI and CKD

#### Restoration of Klotho levels

The predominant focus of studies on MSC-based interventions targeting cellular senescence in AKI has revolved around the involvement of Klotho. According to Condor et al., Wharton’s Jelly-derived MSCs could decrease NF-κB levels and increase Klotho expression compared to the adipose-derived MSC (AD-MSCs) during cell therapy against sepsis-induced AKI [166]. Beyond the protective effect of umbilical cord-derived MSCs (UC-MSCs) in the acute phase, the anti-senescence properties of MSCs protect kidney cells against a maladaptive repair in the long term [167]. Likewise, Klotho gene-modified BM-MSCs could inhibit the Wnt-β/catenin pathway in TECs, and increase their proliferative and immuno-regulation capacities, suggesting a superior choice for cell therapy afterward I/R-induced AKI [168]. Klotho-modified BM-MSCs, by regulating the Klotho/FOXO1 axis and inhibiting downstream cellular oxidative stress, provide greater renal protection compared to the group receiving normal BM-MSC therapy [169]. Moreover, it is reported that Klotho expression in TECs recovering from I/R injury was restored after treatment with Klotho gene-modified BM-MSCs. Therefore, it can be concluded that the partial restoration of Klotho levels through MSC intervention has shown potential benefits for treating AKI.

#### Anti-inflammatory effects

To evaluate the anti-senescence impacts of MSCs in the long term, premature kidney senescence was induced 48 h after I/R damage in an AKI model. The administration of UC-MSCs intraperitoneally (6 h after I/R damage) could lessen oxidative stress and inflammatory responses and reduce the levels of senescence-related proteins by an increased expression of Klotho [167]. Selective targeting of damaged kidneys by MSCs coated by anti-KIM1 (a marker of renal injury) antibody could decrease senescence markers and enhance tubular injury and renal function, acting as a senolytic agent in murine renal artery stenosis (RAS) models [170]. Moreover, senolytic efficacy of AD-MSCs therapy was observed in RAS-induced senescence in human and mouse kidneys. This therapy could improve kidney inflammation, fibrosis, function, and capillary density [171].

#### Angiogenesis effects

miRNA and/or mRNA in MSC-EVs, and angiogenesis factors (HIF-1α and VEGF) can improve mitogenesis in AKI models [172]. The administration of MSC-EVs in an AKI model could increase angiogenesis factors such as angiopoietin, vWF (von Willebrand factor), and CD31 [173]. In an ischemic kidney, kidney-derived MSC-MPs (microparticles) confer renoprotective effects by delivering proangiogenic signals [164]. Moreover, exosomes derived from modified AD-MSC could improve ETCs survival, peritubular capillary loss, and kidney fibrosis, in fibrosis models by activating angiogenesis and SIRT1/eNOS signaling pathway [174]. Likewise, BM-MSC-EVs could improve angiogenesis and kidney regeneration in a cisplatin-induced AKI model by upregulation of SIRT3/eNOS [175]. It is also reported that microvesicles derived from UC-MSCs facilitate tubular epithelial cell growth and dedifferentiation after AKI by transferring hepatocyte growth factor (HGF) mRNA into the damaged tubular cells [176].

#### Anti-fibrotic effects

The mechanism through which MSC-secretome combat fibrosis involves the transmission of their miRNA/mRNA/proteins content that target genes associated with fibrosis such as TGF-β1, tissue inhibitor matrix metalloproteinase 1 (TIMP-1), matrix metalloproteinase (MMP3, 9), snail family transcriptional repressor (SNAI1), collagen I, α-SMA (smooth muscle actin), and PDGFR-β (platelet-derived growth factor receptor β) [177–181]. In herbal nephropathy cases induced by aristolochic acid, BM-MSC-EVs establish a reduction in tubular necrosis and interstitial fibrosis by inhibiting the expression of fibrotic genes like α-SMA, TGF-β1, and collagen Iα1 [182]. Umbilical-MSCs-MVs, enriched with miR-451a, can reverse the EMT in STZ-induced diabetic nephropathy by enhancing E-cadherin expression and reducing fibrosis. The transferred miR-451a acts on the 3UTR regions of cell cycle inhibitors such as P19^INK4d^ and P15^INK4b^, allowing for a resumption of the halted cell cycle and an improvement in the EMT [183]. Through the downregulation of various miRNAs, such as miR21-5p, 34a-5p, 34c-5p, 342-3p, 214-3p, 212-3p and, 132-3p, MSC-EVs can mitigate fibrosis, inflammation, and apoptosis [177, 180, 184]. Moreover, by promoting the activation of Sox9, involved in the repair of damaged TECs, trigger regeneration, and decreases fibrosis [178, 185, 186]. Consequently, EVs have the potential to halt the advancement of tubulointerstitial fibrosis and EMT, thus restoring functionality in CKD.

Moreover, Wang et al. showed that inhibitory effects of MSC-EVs and especially miR-294/miR-133 could prevent aging-related renal fibrosis by blocking the phosphorylation of SMAD2/3 and ERK1/2 and this effect has a negative relationship with aging [187]. Overall, through the regulation of angiogenesis, inflammation, tubular cell de-differentiation and proliferation, inducing autophagy, and decreasing DNA damage, secretome of MSCs can regenerate and rejuvenate renal tissue based on their paracrine pathways.

#### Reducing the progression of senescence-induced aging and diseases

MSCs can decrease cellular senescence and recover renal function in CKD [188]. Human UC-MSCs could protect podocytes in a DN rat model by reducing senescence via the AMPK/mTOR pathway and activating autophagy [189]. These findings suggest that MSC-based therapy can effectively target and ameliorate cellular senescence in CKD, providing a possible therapeutic strategy for the treatment of CKD-related complications.

Evidence shows that MSC-derived secretome are highly effective senotherapeutics, reducing the progression of senescence-induced aging and diseases [190]. MSC-EVs alleviate endothelial cell senescence and induce angiogenesis through miR-146a/Src, accelerating the healing of diabetic and aged mice wounds [191]. Embryonic stem cells (ESCs) and ESC conditioned medium (CM) are considered age-countering interventions with modulatory effects on senescence phenotype. Moreover, Bae et al. found that ESC-CM and its obtained components are senomorphic candidates in a senescence model in human dermal fibroblasts (hDF). ESC-CM drastically resolves the senescent phenotypes of HUVECs and hDFs through a PDGF/FGF-regulated pathway and accelerates wound-healing capacity in a mouse model [192]. Furthermore, ESCs secrete miR-291a-3p-containing exosomes that reduce senescence phenotypes via a TGF-β receptor 2-p21 pathway and improve wound-healing in elder mice [193]. Through inhibiting mTOR and inducing autophagy, MSC-secretome containing 14-3-3ζ and miR-486 can impact senescent kidney cell in STZ-induced type 1 diabetes and cisplatin-triggered AKI models [194–196].

One study revealed that exosome-based therapy significantly declines senescent cells and DNA damage foci through the reduced transcription of senescence elements, SASP, and enhancement of cell proliferation [197]. In addition, the multiple trophic agents obtained from juvenile MSC-derived exosomes are well-designed therapeutic strategies to hypothetically suppress further age-related disorders, which may also be capable of rejuvenation of other tissues and organs [197].

### Challenges

It should be noted that the MSCs’ therapeutic impacts might rely on their senescence status, whether derived from young donors or not [165]. Evidence shows that young donor-derived MSCs are more effective in alleviating kidney aging than older ones [165]. The presence of longer telomeres and some regenerative factors (such as microRNAs) in younger cells may be responsible for their effectiveness. Moreover, it has been revealed that UC-MSCs are effective in decreasing the cell-cycle inhibitors’ expression compared to AD- or BM-MSCs [198]. These results propose that the impacts of MSCs are context and cell-type-dependent. Overall, MSC therapy enhances kidney function, inflammation, and fibrosis and declines IL-6 overexpression and senescence-associated β-Galactosidase activity.

Due to their immunomodulatory and therapeutic potency, MSCs are now the base of cellular and cell-free therapies in the management of many kidney diseases but MSCs are also not immune to aging. Following either the process of aging in older individuals, uremic milieu, or prolonged in vitro expansion, MSCs encounter cellular senescence [199]. It is reported that ROS production, ischemia, inflammation, cellular microenvironment, poor control of the disease, and pathophysiological uremic milieu induced by CKD/AKI can decrease the efficacy of MSC therapy due to premature cellular senescence, contributing to their poor regenerative potential [188, 200]. The senescence of MSCs-induced by CKD/AKI results in the modification of their secretory profile and a decline in their differentiation and proliferation capabilities [201]. The presence of certain senescence markers may also impair the immunomodulatory effect of MSCs, reducing their effectiveness in therapeutic contexts.

Given the failure of cell culture conditions to inverse the MSC phenotype following exposure to uremic circumstances, it is reasonable to hypothesize that epigenetic alterations, resembling those detected in aging MSCs, are prompted by CKD and this could potentially explain what has been referred to as uremic memory based on clinical clarifications. Multiple uremic factors have been identified as potential causes of MSC impairment. P-cresol and indoxyl sulfate have been shown to reduce MSC proliferation in mice. Furthermore, diabetes constructs an adverse microenvironment for MSCs, rendering their survival, and migration to infected tissue, and make functional performance more challenging. MSCs exhibit low viability and proliferation ability, accompanied by decreased glycosaminoglycans and proteoglycans in the surrounding tissues [202]. Advanced glycosylated end-products production initiates apoptosis and ROS generation, further preventing the proliferation of MSCs. Additionally, under hypoxic conditions, oxidative stress adversely affects the paracrine effects of MSCs in diabetic individuals. The elevated levels of superoxide in hypoxic MSCs impede the production of angiogenic growth factors [203, 204]. Moreover, MSCs exhibit impaired migration ability under hyperglycemic conditions [205]. Other factors that promote MSC aging and contribute to damage in stem cells include mitochondrial dysfunction, non-coding RNAs, EVs, genetic abnormality, pro-inflammatory molecules, and angiotensin II, all of which are present in kidney diseases.

Other significant challenges include MSC cell sources, the safety of MSCs, and the development of effective isolation methods. Different sources of MSCs (e.g., adipose tissue, bone marrow) show variability in their functional capabilities and aging characteristics. This heterogeneity complicates the selection of optimal MSC populations for therapy, as older or more senescent cells may not provide the desired regenerative effects. Understanding how these factors influence MSC behavior will provide insights into optimizing MSC-based therapies for kidney diseases.

### MSC rejuvenation strategies

There is a significant demand for the development of innovative research endeavors aimed at deepening the understanding of cellular senescence and facilitating the discovery of novel approaches to counteract senescence under pathophysiological contexts. Preconditioning of MSCs prolongs their lifespan and expands their functionality. Kim et al. suggested that metformin preconditioning MSCs could improve vessel repair and functional recovery, proliferative potential of MSCs, and inhibit CKD-induced DNA damage and senescence of MSCs. Pretreatment with metformin was found to mitigate oxidative stress and senescence in an ischemic disease model associated with CKD [206]. Metformin acts on MSCs by activating AMP-activated protein kinase (AMPK), reducing oxidative stress, modulating inflammatory pathways, and inducing autophagy, thereby maintaining cellular homeostasis and enhancing MSC longevity. Likewise, melatonin can defend CKD-MSCs against senescence by improving mitochondrial function, cell proliferation, glycolytic metabolism [207] and their regenerative potential [208]. Melatonin regulates MSCs via paracrine mechanisms, controls ROS generation, promotes cell proliferation by inducing the expression of SRY-box transcription factor 2, and modulates immune responses, all contributing to enhanced MSC survival and function. Additionally, MSCs treated with fucoidan can augment angiogenesis, regeneration, and cell proliferation in an ischemia CKD model [209]. Fucoidan exerts its effects through strong antioxidant properties, modulation of inflammatory pathways, and promotion of MSC proliferation and differentiation. Preconditioning with *Tinospora cordifolia* and *Withania somnifera* (herbal extracts) has been found to delay senescence in Wharton’s jelly MSCs [210]. These pre-conditioning strategies may contribute to maintaining the regenerative potential of MSCs and delaying the onset of senescence.

Rejuvenating MSCs can reverse aging-associated phenotypes as it is a result of cellular senescence. Preconditioning with miscellaneous pharmacological compounds, mTOR inhibitors, antioxidants, resveratrol, and Metformin can enhance the stemness and therapeutic potency of MSCs [211, 212]. Hypoxic and Serum-free medium preconditioning rejuvenates MSCs and their secreted profile [213] and it can be applied in targeting kidney senescent cells under physiological and pathological circumstances.

#### Clinical implications and future directions

To the best of our knowledge, no registered clinical trial proceeds for the treatment of kidney diseases using MSC-derived factors on clinicaltrials.gov. However, a list of completed and ongoing clinical trials is provided that study the therapeutic potential of MSCs for diverse kidney conditions (Tables 2 and 3).

**Table 2:** Completed randomized clinical trials using MSC therapies for kidney diseases

No.	Trial ID	Disease	Country	Phase	Study type	Source of MSCs	Autologous/ allogeneic	Number of patients	Administration frequency	Duration	Outcomes	Ref.
1	NCT02166489	ADPKD	Iran	I	Single-arm phase	BM-MSCs	Autologous	27	Cultured MSCs (1–2 × 10^6^/kg) through the cubital vein	12 months	Adverse events, safety and tolerability Renal function	[214]
2	NCT03174587	LN	Republic of Korea	I	Nonrandomized, open-label, single-arm	BM-MSCs	Allogeneic	7	2.0 × 10^6^ cells/kg and escalated to 3.0 × 10^6^ cells/kg by IV infusion at a rate of 1 mL/min	28 days	Adverse events, safety, tolerability	[215]
3	NCT02195323	DN	Iran	I	Randomized, single-arm, double-blind, controlled	BM-MSCs	Autologous	7	IV infusion (1–2 × 10^6^ cells/kg) of cultured MSCs	18 months	Adverse events, safety and tolerability Renal function	[216]
4	NCT01843387	LN	Australia	I	Randomized, Placebo-controlled, Dose Escalation	BM-MSCs	Allogeneic	30	single IV infusion of allogeneic MPC 150 × 10^6^, 300 × 106, or placebo	12 weeks	Adverse events, safety, efficacy, and tolerability Renal function	[217]
5	NCT02585622	DN	Ireland, Italy, and United Kingdom	Ib/IIa	Randomized, double-blind, placebo-controlled	BM-MSCs	Allogeneic	16	IV infusion of MSC (80 × 10^6^ cells) or placebo	18 months	Safety, tolerability, and efficacy	[218]
6	-	DN	Kazakhstan	-	Open-label pilot study	BM-mononuclear stem cells	Autologous	15	~ 140 × 10^6^ cells (in average 88 × 10^6^) diluted in 200 ml saline via IV infusion (50 ml/hour)	6 months	Urinary markers, MAU, urinary type-IV collagen, and urinary uNGAL	[219]
7	NCT01539902	LN	China	-	Prospective, randomized, double-blind, placebo-controlled	hUC-MSCs	Allogeneic	18	MSC (2 × 10^8^ cells) or placebo	12 months	Adverse events, time to remission, proteinuria, SCr, SLEDAI and BILAG scores, ANA, Serum C3 and C4	[220]

*ADPKD* Autosomal Dominant Polycystic Kidney Disease, *ANA* Antinuclear Antibody, *BILAG* British Isles Lupus Assessment Group, *BM-MSC* Bone marrow-derived mesenchymal stem cells, *DN* Diabetic nephropathy, *eGFR* Estimated glomerular filtration rate, *hUC-MSC* Human umbilical cord-derived mesenchymal stem cells, *IV* Intravenous, *LN* Lupus nephritis, *MAU* microalbuminuria, *SCr* Serum creatinine, *SLEDAI* Systemic Lupus Erythematosus Disease Activity Index, *uNGAL* Urinary neutrophil gelatinase-associated lipocalin

**Table 3:** Ongoing clinical trials using MSC therapies for kidney diseases

No.	NCT Number	Study Status	Disease	Interventions	Primary Outcome Measures	Follow-up	Phases	Enrollment	Locations
1	NCT04194671	UNKNOWN	AKI	MSCs	Renal function (SCr)	28 days	I/II	80	China
2	NCT01602328	TERMINATED	AKI	AC607 (BM-MSCs)	SCr	30 days	II	156	USA
	NCT03015623	UNKNOWN	AKI	SBI-101 (MSCs)	Safety and tolerability, adverse events, Outcomes	180 days	I/II	24	USA
3	NCT06654193	NOT_YET_RECRUITING	AKI	Allogeneic HB-AD-MSCs	Adverse events, Duration of AKI at Stage 2 or higher, Proportion of patients with a duration of Stage 2 AKI more than 2 days	1 year	I/II	70	USA
4	NCT04388761	WITHDRAWN	IRI	Allogeneic AD-MSCs	Adverse events, control bleeding, sub-scapular kidney hematoma or arteriovenous fistula formation, and development of stroke, myocardial infarction, or pulmonary embolism	1 year	I	0	USA
5	NCT01840540	COMPLETED	Ischemic Nephropathy	Arterial infusion of autologous MSCs	Renal blood flow and function	2 years	I	6	USA
6	NCT03840343	TERMINATED	DN	Autologous AD-MSCs	Adverse Events	15 months	I	2	USA
7	NCT04216849	UNKNOWN	DN	hUC-MSCs	UACR, urinary albumin SCr ratio	48 weeks	I/II	54	China
8	NCT04125329	UNKNOWN	DN	hUC-MSCs	Adverse Events	60 weeks	Early I	15	China
9	NCT03288571	UNKNOWN	DN	WJ-MSCs	Adverse Events, Safety and Tolerability	6 months	I/II	20	Jordan
10	NCT04562025	UNKNOWN	DN	UC-MSCs	Adverse Events	48 weeks	N/A	38	China
11	NCT05362786	COMPLETED	CKD	Allogeneic AD-MSCs	Adverse events and eGFR	15 months	I	14	USA
12	NCT04869761	ACTIVE_NOT_RECRUITING	CKD	Allogeneic AD-MSCs-Single Infusion/Two Infusions	Adverse events	22 months	I	10	USA
13	NCT04592640	UNKNOWN	CKD	hAM-MSCs	Wound Healing	Up to 1 year	N/A	7	China
14	NCT02266394	COMPLETED	CKD	MSC delivery with stent placement	Kidney function, Renal Tissue oxygenation, Safety, tissue injury markers	2 years	I	42	USA
15	NCT03321942	UNKNOWN	CKD	AD-MSCs	SCr, intravenous blood sampling	3 months	N/A	100	China
16	NCT05018845	RECRUITING	CKD	Allogenic UC-MSCs	Safety (adverse events)	4 years	I	20	Antigua and Barbuda
17	NCT05512988	UNKNOWN	CKD	UC-MSCs	GFR, Adverse Events	13 months	I/II	44	China
18	NCT02195323	COMPLETED	CKD	IV injection of 2 × 10^6^/kg autologous MSCs	Mass formation, Creatinine	6 months	I	7	Iran
19	NCT03939741	RECRUITING	CKD	SVF (Autologous Non Expanded AD-MSCs)	Adverse events, GFR and split renal function (DTPA Renogram, SCr), Need for dialysis is described	48 weeks	I/II	31	Bangladesh
20	NCT04998461	NOT_YET_RECRUITING	CKD	hUC-MSCs	Comparison of gene expression in Urinary Stem Cells	1 day	-	60	France
21	NCT05042206	COMPLETED	CKD	allogenic BM-MSCs	Adverse events	12 months	I	10	Korea
22	NCT02166489	COMPLETED	Chronic Renal Failure	IV injection of autologous MSCs	Mass formation	1 month	I	6	Iran
23	NCT00659217	UNKNOWN	LN	Autologous MSC transplantation	The proportion of participants who achieve and maintain remission	5 months	I/II	20	China
24	NCT04318600	COMPLETED	LN	hAM-MSCs	Adverse Events	60 weeks	I	16	China
25	NCT03673748	RECRUITING	LN	Allogenic MSCs	renal function (GFR, proteinuria, inactive sediment, RBCs, leukocytes, absence of RBC casts, and serum albumin)	24 weeks	II	20	Spain
26	NCT03580291	UNKNOWN	LN	MSCs	Total remission rate	24 weeks	II	230	China
27	NCT06485648	NOT_YET_RECRUITING	LN	UC-MSCs	Proteinuria, serum creatinine, kidney function	12 months	Early I	96	China
28	NCT03458156	UNKNOWN	LN	UC-MSCs	SLEDAI-2000 score	12 months	N/A	30	China
29	NCT05631717	RECRUITING	LN	hUC-MSCs	Response rates (serum creatinine, proteinuria)	24 Weeks	III	40	China
30	NCT03174587	COMPLETED	LN	Allogenic BM-MSCs	Adverse events, Laboratory test(hematology/blood chemistry, urine test), Vital signs, Physical examination, ECG	28 days	I	7	Korea
31	NCT01539902	UNKNOWN	LN	hU-CMSCs	Efficacy and Safety, renal function (Urinary RBC, proteinuria	6 months	II	25	China
32	NCT06058078	RECRUITING	LN	RY_SW01 cell (allogenic UC-MSCs) injection	Adverse Events, renal function	24 weeks	II	60	China
33	NCT04522505	COMPLETED	LN	CS20AT04-LN101-E (Allogenic BM-MSCs)	Adverse events, Incidence of abnormal results of Physical examination, Hematology tests, Chemistry tests, Urine analysis	57 months	-	6	Korea
34	NCT02490020	UNKNOWN	Kidney Transplant	MSCs	Incident rates of BPAR and DGF, renal function, renal biopsy, and other opportunistic infection	1 year	I	260	China
35	NCT04445220	UNKNOWN	Kidney Transplant	SBI-101 (MSCs)	Safety, tolerability, adverse events	180 days	I/II	22	USA
36	NCT02561767	UNKNOWN	Kidney Transplant	BM-MSCs	eGFR	1 month	I/II	120	China
37	NCT02563366	UNKNOWN	Kidney Transplant	BM-MSCs	eGFR	1 month	I/II	120	China
38	NCT00659620	UNKNOWN	Kidney Transplant	MSCs	SCr and SCr clearance rate	5 months	I/II	20	China
39	NCT04342325	COMPLETED	IgAN	infusion of ADR-001 (MSCs)	Adverse events	6 weeks	I	9	Japan
40	NCT02382874	UNKNOWN	FSGS	IV injection of allogenic AD-MSCs	Liver function, SCr, Proteinuria	2 weeks	I	5	Iran
41	NCT02966717	UNKNOWN	Nephrotic Syndrome	MSCs	SCr, The percentage of ESRD or death	3 years	II	116	China
42	NCT02492490	UNKNOWN	Uremia	Autologous SVF-derived MSC transplantation	reducing the dosage of CNI by 30% in Kidney Transplantation	1 year	I/II	120	China
43	NCT02808208	ACTIVE_NOT_RECRUITING	ESRD	Single Application/Two Application of AD-MSCs	Hemodialysis outflow vein diameter	12 months	I/II	74	USA
44	NCT04392206	RECRUITING	ESRD	AD-MSCs	Adverse events, safety by inflammation, infection (local or systemic), aneurysm formation, clinically significant increase or decrease in blood flow or thrombosis formation	12 months	I	15	USA
45	NCT01429038	COMPLETED	Kidney Failure	MSCs	Infusional toxicity, Incidence, timing and severity of any clinical complication, Incidence of infections and cancers	Over 2 years	I/II	40	Belgium

Adapted from www.clinicaltrials.gov, *AD-MSCs* Adipose derived-mesenchymal stem cells, *AKI* acute kidney injury, *BM-MSC* Bone marrow-derived mesenchymal stem cells, *BPAR* Biopsy proven acute rejection, *CKD* Chronic kidney disease, *CNI* Calcineurin inhibitors, *DGF* Delayed graft function, *DN* Diabetic nephropathy, *DPTA* Diethylenetriamine Pentaacetic Acid, *eGFR* Estimated glomerular filtration rate, *ESRD* end-stage renal disease, *IgAN* IgA nephropathy, *FSGS* focal segmental glomerulosclerosis, *HB * Hope Biosciences, *hAM-MSCs* Human amniotic membrane-derived mesenchymal stem cells, *hUC-MSC* Human umbilical cord-derived mesenchymal stem cells, *IV* Intravenous, *LN* Lupus nephritis, *SCr* Serum creatinine, *SLEDAI* Systemic Lupus Erythematosus Disease Activity Index, *SVF* Stromal vascular fraction, *WJ-MSC* Wharton's jelly-derived mesenchymal stem cells

## Conclusions

In the kidney, senescence may have beneficial effects on injury progression and recovery, but it can also contribute to the development of AKI, CKD, and the rejection of transplanted organs. This is evident in the heightened presence of cyclin-dependent kinase inhibitors, oxidative stress, reduced klotho expression, and the shortening of telomeres. Various treatments aimed at targeting senescence have exhibited the ability to prolong lifespan and mitigate kidney damage in a variety of animal models. The therapeutic nature of MSC secretome in renal senescence reveals its capacity as a cell-free senotherapy agent. Furthermore, genetic modification or the engineering for overexpression/inhibition of specific microRNAs and proteins along with certain preconditioning stimuli can influence the quantity and quality of secreted factors. Since human MSCs are amplifiable in bioreactors to produce great yields of MSC-derived secretome, it can be a promising therapeutic strategy for age-related renal disorders. Initial clinical trials have promising in applying therapeutic methods focused on senescence that could prove advantageous in addressing human ailments. While early clinical trials suggest that MSC- EV therapies hold promise for kidney diseases, the use of EVs for kidney senescence needs further optimization, including determining the appropriate dosage and treatment intervals. However, it is imperative to distinguish between harmful and protective senescence, a task that necessitates further investigation in animal models and clinical trials. Substantial clinical trials are required to implement these strategies effectively in patient care.

## Data Availability

This is a review article, no new data were created or analyzed in this study.

## Abbreviations

AMPK: AMP-activated protein kinase
ATM: Ataxia-telangiectasia mutated
ATP: Adenosine triphosphate
C/EBPα: CCAAT/enhancer-binding protein alpha
CKD: Chronic kidney disease
CM: Conditioned medium
CTGF: Connective tissue growth factor
DPP4: Dipeptidyl peptidase 4
EC: Endothelial Cell
EMT: Epithelial–mesenchymal transition
ESCs: Embryonic stem cells
ETC: Electron transport chain
GBM: Glomerular basement membrane
GFR: Glomerular filtration rate
GRO-α: Growth regulated protein alpha
GSK 3B: Glycogen Synthase Kinase 3 Beta
hDF: Human dermal fibroblasts
HGF: Hepatocyte growth factor
HGPS: Hutchitson-Gilford progeria syndrome
IGF-1: Insulin-like growth factor-1
JAK: Janus kinase
MCP-1: Monocyte chemoattractant protein-1
MMP: Matrix metalloproteinase
MQ: Macrophage
mTOR: Mammalian target of rapamycin
MuSCs: Muscle stem cells
NF-κB: Nuclear factor kappa-light-chain-enhancer of activated B cells
PAI-1: Plasminogen activator inhibitor-1
PD-1: Programmed cell death protein 1
PDGFR-β: Platelet-derived growth factor receptor β
PEC: Parietal Epithelial Cell
PGC-1α: Peroxisome proliferator–activated receptor gamma coactivator-1 alpha
RAAS: Renin-angiotensin-aldosterone system
RBF: Renal blood flow
ROS: Reactive oxygen species
SASP: Senescence-associated secretory phenotype
siRNA: Small interfering RNA
SIRT1: Sirtuin-1
SNAI1: Snail family transcriptional repressor 1
TCA: Tricarboxylic acid
TEC: Tubular epithelial cells
TGF-β: Transforming growth factor beta
Grhl2: Grainyhead-like 2
TIMP-1: Tissue inhibitor matrix metalloproteinase 1
TNF- α: Tumor necrosis factor alpha
UUO: Unilateral Ureteral Obstruction
α-SMA: Smooth muscle actin
